# Importance of Angiogenin and Endothelial Progenitor Cells After Rehabilitation Both in Ischemic Stroke Patients and in a Mouse Model of Cerebral Ischemia

**DOI:** 10.3389/fneur.2018.00508

**Published:** 2018-06-29

**Authors:** Marina Gabriel-Salazar, Anna Morancho, Susana Rodriguez, Xavi Buxó, Nicolás García-Rodríguez, Guillem Colell, Albert Fernandez, Dolors Giralt, Alejandro Bustamante, Joan Montaner, Anna Rosell

**Affiliations:** ^1^Neurovascular Research Laboratory and Neurology Department, Vall d'Hebron Research Institute, Universitat Autònoma de Barcelona, Barcelona, Spain; ^2^Unidad de Rehabilitación Neurológica y Daño Cerebral, Hospital Vall d'Hebron, Barcelona, Spain

**Keywords:** stroke, rehabilitation, angiogenesis, angiogenin, endothelial progenitor cell, biomarker

## Abstract

**Background:** Rehabilitation therapy is the only available treatment for stroke survivors presenting neurological deficits; however, the underlying molecules and mechanisms associated with functional/motor improvement during rehabilitation are poorly understood.

**Objective:** Our aim is to study the modulation of angiogenin and endothelial progenitor cells (EPCs) as repair-associated factors in a cohort of stroke patients and mouse models of rehabilitation after cerebral ischemia.

**Methods:** The clinical study included 18 ischemic strokes admitted to an intensive rehabilitation therapy (IRT) unit, 18 non-ischemic controls and brain samples from three deceased patients. Angiogenin and EPCs were measured in blood obtained before and up to 6 months after IRT together with an extensive evaluation of the motor/functional status. In parallel, C57BL/6 mice underwent middle cerebral artery occlusion, and the pasta matrix reaching-task or treadmill exercises were used as rehabilitation models. Angiogenin RNA expression was measured after 2 or 12 days of treatment together with cell counts from EPCs cultures.

**Results:** Brain angiogenin was identified in both human and mouse tissue, whereas serum levels increased after 1 month of IRT in association with motor/functional improvement. EPC populations were increased after stroke and remained elevated during follow-up after IRT. The mouse model of rehabilitation by the task-specific pasta matrix exercise increased the number of EPCs at 2 days and increased angiogenin expression after 12 days of rehabilitation.

**Conclusions:** Angiogenin and EPCs are modulated by rehabilitation after cerebral ischemia, suggesting that both angiogenin and EPCs could serve as biomarkers of improvement during rehabilitation or future therapeutic targets.

## Introduction

Stroke is one of the leading causes of death and long-term disability worldwide and leads to 5 million people becoming permanently disabled annually (1–3). Even with the new advances in diagnosis and therapeutic options that are available in the acute phase of a stroke (4), the only approved treatment in subacute and chronic phases is neurorehabilitation to reduce stroke-related disability, thereby leading to an improved quality of life and independence in daily living activities (5).

Rehabilitation after stroke needs an inter-disciplinary care-team including physiotherapists, occupational therapists, language, and speech therapists, working under the direct supervision of a physiatrist, who might be assisted in medical decisions by the use of biomarkers monitoring the neurorepair process (6). The use of clinical measures, physiological parameters, or neuroimaging biomarkers to predict long-term motor recovery in the context of rehabilitation has been studied in recent years (7, 8), but minor contributions have been achieved for molecular biomarkers. It has been described that the improvements in neurological function during rehabilitation respond to repair or compensatory mechanisms that induce plasticity changes involving multiple molecular pathways (9). By identifying these pathways and bio-molecules, we predict that personalization of rehabilitation treatments may be possible. In this regard, some studies have positively shown associations between the levels of oxidative stress markers, neurotransmitters and proteases in biological fluids, and motor function in stroke patients undergoing rehabilitation programs (10–13).

Angiogenesis and vascular remodeling are mechanisms activated early after stroke which remain elevated from days to several weeks as a response to increased collateral blood supply and tightly coupled to neurogenesis and oligodendrogenesis as part of the endogenous neurorepair response (14–16). Moreover, angiogenesis has been associated with a neurological improvement in animal models of stroke (17–19) and modulated by physical exercise (17, 20). Many angiogenesis-related molecules (both promoters and inhibitors) have been described to participate in angiogenesis in the context of stroke, but less is known about their regulation during rehabilitation. In the present study, we focused on two well-known angiogenesis mediators: a molecular player (angiogenin) and a cellular source which sustains angio-vasculogenesis (endothelial progenitor cells, EPCs); both with unknown role as responders to rehabilitation therapy after stroke. Angiogenin has been widely associated with angiogenesis in cancer disease, participating in cell proliferation, migration, and invasion of endothelial cells (21). Its expression has been identified also in neurons and as part of the secretome of EPCs (22, 23). On the other hand, EPCs are well-known mediators of angio-vasculogenesis and vascular remodeling in the adulthood (24, 25). After stroke, circulating EPCs increase in blood and have been identified as markers of infarct size, neurological status, and functional outcome (26, 27).

Our hypothesis is that angiogenin and EPCs are modulated during rehabilitation after cerebral ischemia serving as biomarkers of functional/motor outcome related to their participation in plasticity mechanisms during neurorepair. To test this hypothesis, our study combines a cohort of stroke subjects under intensive rehabilitation therapy (IRT) in which angiogenin and circulating EPCs levels are analyzed. Additionally, the neurological status is monitored in patients using a battery of tests during a 6-month period. Further, two rehabilitation models in mice after cerebral ischemia (task-specific based exercise or physical exercise) are used, where brain and circulating angiogenin determinations together with EPCs cultures are analyzed.

## Materials and methods

### Study cohorts and rehabilitation intervention

Stroke and control cohorts are part of the Studying Markers of Angiogenesis during Rehabilitation Therapy after Stroke (SMARTS) study (13). In the current investigation, ischemic stroke patients were prospectively admitted n a neurorehabilitation unit from February 2014 to May 2015. Inclusion criteria were as follows: first-ever ischemic stroke, age < 70 years, somatosensory or ataxic hemiparesis, time until start of IRT < 3 weeks after stroke, stable medical condition, and endurance to participate for a minimum of 3 h/day in a therapy program. Exclusion criteria were malignant infarctions, sensory or global aphasias, cognitive deficits (Mini-Mental State Examination score < 23), terminal illness, inflammatory or infectious diseases, or previous deficits of the upper/lower limb. A total of 71 patients with stroke received IRT in our hospital; however, only 18 met the inclusion criteria of our study. The excluded patients presented with hemorrhagic stroke (*n* = 24), malignant infarction (*n* = 13), global aphasia (*n* = 10), human immunodeficiency virus infection (*n* = 1), or carotid tumor (*n* = 1), time to IRT > 3 weeks (*n* = 1), or denied the informed consent (*n* = 3). The included patients followed a comprehensive intensive rehabilitation program, including physiotherapy, occupational therapy, speech therapy, and/or neuropsychology, if required, for a minimum of 3 h/day and 5 days/week as recommended by the clinical practice guidelines for the management of stroke in our health care system (28).

The protocol started with a physiatrist assessing patient's deficits within the first 48 h after stroke and designing an initial rehabilitation program. Passive mobilizations performed in bed started within the first 24 h after stroke if there were no medical conditions that contraindicate it, and at 48 h patients started a moderate-intensity rehabilitation program. Those patients who presented severe or moderate deficits in ≥2 functional areas and who met the inclusion criteria for an intensive rehabilitation program were transferred to the neurorehabilitation unit for inpatient IRT, whereas those patients who were able to walk with some support/assistance were released to their household and started the daily IRT at the day hospital of the neurorehabilitation unit. IRT continued until completion of a minimum of 75% of the proposed objectives or when functional stability was achieved. If there were further objectives, the patient continued a high- to moderate-intensity outpatient rehabilitation program.

In parallel, 18 non-ischemic healthy individuals without any known neurological, malignant, infectious, or inflammatory diseases volunteered to participate as the non-stroke cohort; a portion of the individuals were subjects with hypertension studied in the Investigating Silent Strokes in Hypertensives Study (29) or patient relatives (spouse) who volunteered.

The study protocol was approved by the local clinical research ethics committee (Num. 317/2013), and all patients/healthy volunteers signed the corresponding informed consent in accordance with the Declaration of Helsinki.

### Clinical study protocol

**Figure 1:**
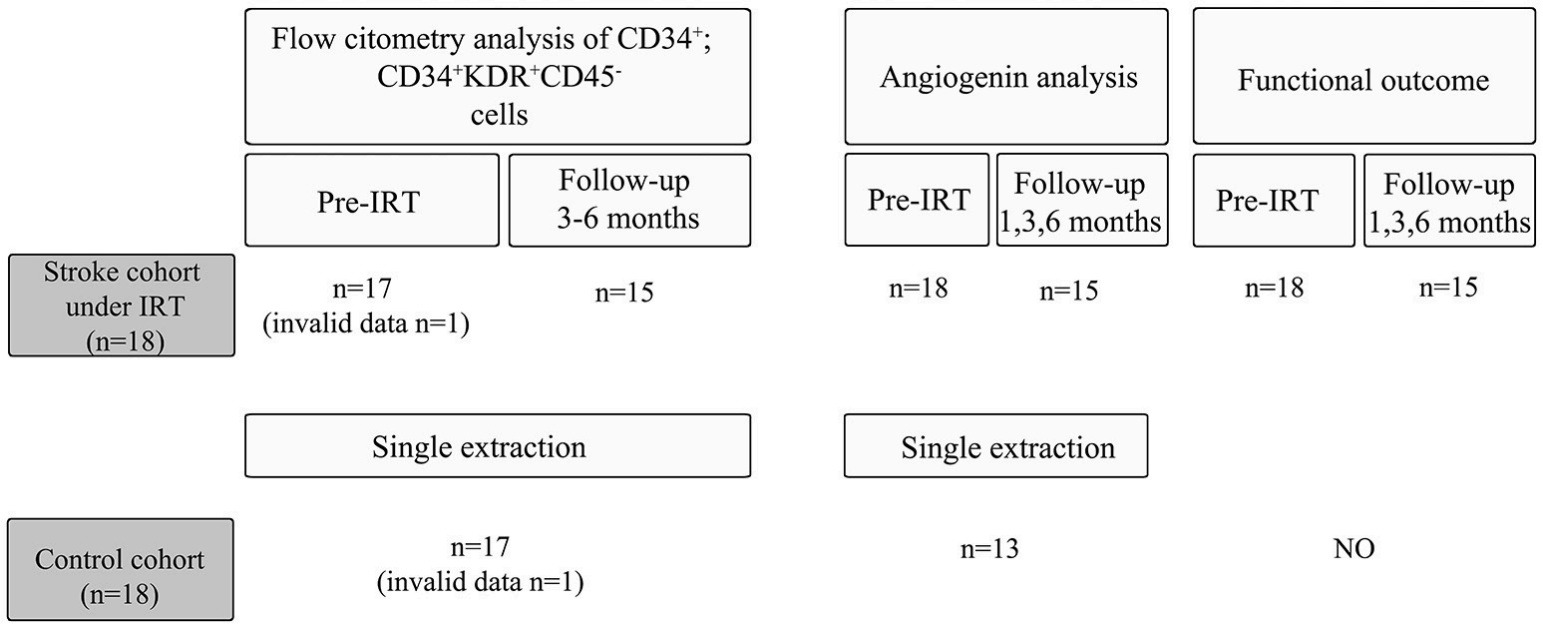
Study overview for the control and the stroke cohort.

Of the 18 included stroke patients, 1 patient was excluded during the follow-up after having a recurrent stroke, whereas 2 other patients chose to withdraw from the study during the follow-up visits (Figure 1). Data related to subject demographics, risk factors, medications, comorbidities, exercise, and clinical stroke characteristics were obtained from both cohorts by researchers blinded to the angiogenin and EPC determinations. Specific visits were performed by an experienced physiatrist at baseline prior to starting the IRT and at 1, 3, and 6 months after starting the therapy program. During these visits, motor function, and functional status were assessed as described below, and peripheral blood was extracted in serum-separating tubes and centrifuged at 1,500 × g for 15 min to obtain serum, which was stored at −80°C, and in EDTA tubes for EPC counts as described below.

### Infarct volume evaluation and functional follow-up

Infarct volume was evaluated in follow-up CT (computed tomography) scans performed 24–48 h after stroke by an experienced neurologist, blinded to clinical information, angiogenin levels, and cell populations of interest. Infarct volume was measured according to the formula AxBxC/2, where A and B represent the largest perpendicular diameters through the hypodense area on CT scan, and C represents the thickness of the infarct (30).

Baseline and follow-up visits included different functional/motor tests: the modified Rankin scale (scores 0–6), the Granger modified Barthel Index (BI) (scores 0–100) (31) and dependence categories (32), the Fugl-Meyer Assessment (FMA) score for the upper extremity (scores 0–66), the Functional Ambulation Categories (FAC) (scores 0–5), the Chedoke Arm and Hand Activity Inventory (scores 13–91) (33), the 10-m walk test, and the Medical Research Council (MRC) scale (scores 0–5) of the disabled hemisphere (upper and lower extremities at the proximal/distal level).

Improvement classifications were obtained after comparing the scores during follow-up visits vs. baseline scores: Rankin improvement was defined as a decrease of ≥1 points. For the FMA, improvement was defined as an increase ≥10 points, described previously as the minimal clinical important difference (34). For the Chedoke Arm and Hand Activity Inventory, an improvement was defined as an increase of ≥7 points (35). For the 10-m walk test, the walking velocity was calculated, and improvement was considered if walking velocity increased by > 0.3 m/s (36). The FAC was categorized into three categories: cannot walk (score 0), dependent walk (scores 1–3), and independent walk (scores 4 and 5). Improvement was defined as a shift to an upper category (37). For the MRC scale, our analysis differentiated between normal (score 5) or impaired (scores 0–4) muscle strength.

### Animal habituation

A total of 88 C57BL/6 mice (males between 6 and 12 weeks) purchased from Janvier laboratories (Saint Berthevin, France) were used in this study. The mice were housed in groups of 3–5 animals in a temperature/humidity-controlled room and maintained on a 12-h light-dark cycle. All animals were given water and food *ad libitum*, but 7 days before habituation and throughout the entire experimental procedure, food was restricted to 2–2.5 g/animal/day of chow pellets in order to increase the motivation for the specific task, while weight was controlled during the entire procedure. Experimental procedures were approved by the Ethics Committee of Animal Experimentation of the Vall d'Hebron Research Institute (protocol number 21.16) and were conducted in accordance with Spanish legislation and the Directives of the European Union.

**Figure 2:**
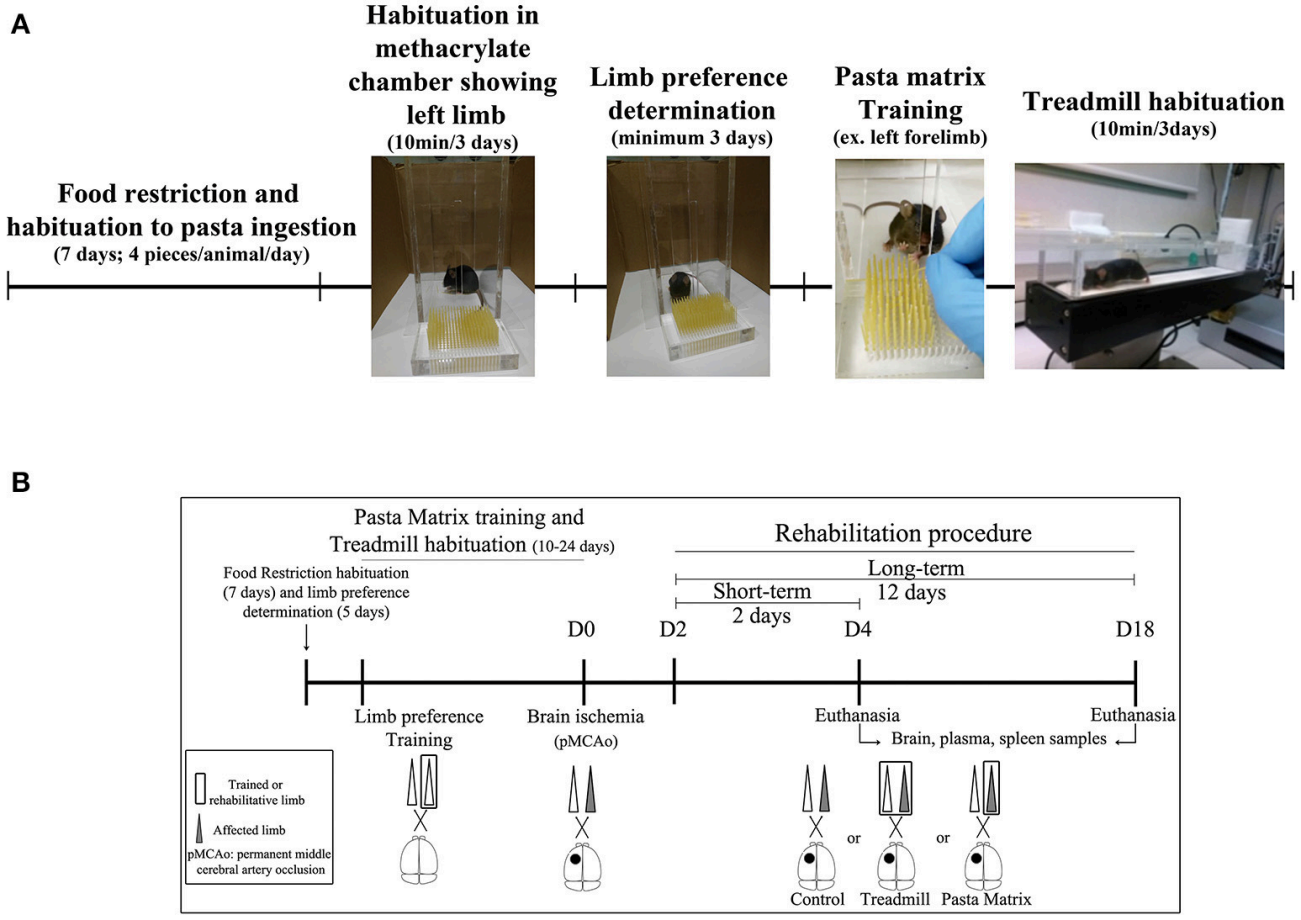
Experimental mice procedure. **(A)** Scheme of animal habituation and training to pasta matrix and treadmill rehabilitation protocols. **(B)** The experimental design overview: from food restriction and pasta matrix training/habituation to cerebral ischemia and rehabilitation protocols. D, day.

All animals were habituated and trained on the pasta matrix reaching task with a protocol adapted from Kerr et al. (38) or habituated to the treadmill apparatus. In brief all 74 mice included in the study were offered with four pieces of uncooked pasta per animal (1.6 cm each, Capellini pasta DeCecco) and placed in their housing-cages during 7 consecutive days. To avoid neophofic responses mice were simultaneously habituated to the methacrylate chamber (20 × 15 × 8.5 cm with two apertures) with small pasta pieces on the floor and the filled pasta matrix structure in front of one of the apertures for three consecutive days (10 min/day); see Figure 2A.

The habituation protocol for the treadmill was conducted after the pasta matrix training described below. Briefly, from 3:00 to 6:00 p.m., all mice were placed in a treadmill apparatus 10 min/day for 3 consecutive days, to avoid posterior stress responses from a new environment during rehabilitation.

### Forelimb laterality and pasta-matrix training

After the pasta matrix habituation, limb preference was established by testing the mice for a minimum of 3 days between 9:00 a.m. to 1:00 p.m. Mice were placed in the testing chamber with a matrix full of pasta in the front, and they were encouraged to reach pasta pieces from the aperture for 10 min or a maximum 10 attempts. The number of attempts with the right or the left forelimb was recorded and the limb preference was determined by the 70% of limb dominance. After individual laterality preference was established, mice were trained for their preferred forelimb by filling only half of the matrix with pasta (contralateral of the preferred limb; Figure 2A). All animals performed at least 10 days of training (5 days/week) consisting on 100 attempts or 15 min in the testing chamber. The training was finished the day each mice was able to break a minimum of nine pieces of pasta consistently for a minimum of 3 consecutive days.

### Permanent focal cerebral ischemia model

Permanent cerebral ischemia was performed on 84 mice as previously described (39) by the permanent distal occlusion of the middle cerebral artery (pMCAo), depending on the animal limb preference, while four other mice received sham surgery. From them, 14 animals were excluded after applying the following criteria: surgical bleedings (*n* = 5), death during surgery (*n* = 3), and infarct volume < 5 mm^3^ (*n* = 6, when this was evaluated and regardless the treatment group).

Animals were anesthetized with isofluorane (Abbot Laboratories, Spain) for a maximum period of 30 min via face-mask (4% for induction and 1–2% for maintenance in Medicinal Air, 79%N_2_/21%O_2_) and body temperature (36.5–37°C) was controlled by a probe thermometer with mice laying on a heating pad. Mice eyes were protected from corneal damages during surgery using an ophthalmic lubricating ointment (Lipolac^TM^, Angelini Farmaceutica, Spain). An incision was made between the left eye and ear under an operating microscope (Leica MS5; Leica, Heerburg, Switzerland) and the temporal muscle was cut and divided exposing the left lateral part of the skull. The MCA was identified through the semi-translucent skull, and a small burr hole (2–3 mm diameter) was made using a high-speed microdrill at the level of the inferior cerebral vein to expose the M1 portion as described (39). Saline was applied to the area throughout the procedure to prevent heat injury, keeping the area always hydrated. Then, the MCA was compressed using a micromanipulator and indirectly electrocauterized by heating the 30-G needle compressing the MCA. Cerebral blood flow (CBF) was monitored using a Laser-Doppler flowmetry (Moor Instruments, UK) with a probe inserted distally to the cauterization site, only animals with a reduction in CBF to below 80% were included. Buprenorphine (0.05–0.1 mg/Kg) was administered subcutaneously after the surgery, the temporal muscle was placed to the original position, skin sutured with a silk suture and mice were allowed to spontaneously recover from anesthesia. All sham animals were operated with the same surgical procedure with the exception of the pMCA occlusion (pMCAo). After 4 or 18 days of ischemia (corresponding to 2 or 12 days of rehabilitation) or after 4 days of surgery in the sham group, animals were euthanized following specific procedures described below.

### Pre-clinical rehabilitation models

Rehabilitation treatments began 48 h after pMCAo for all animals. Mice were randomly allocated to the three experimental groups using a computer-generated randomization list: No-Rehabilitation (No-RHB); Pasta Matrix (PM), where the reaching-task consisted of performing 100 attempts with the affected limb inside the chamber; and Treadmill, where mice received 30 min of exercise by increasing the speed every 10 min (10, 15, and 20 cm/s) without any aversive stimulus (such as the electric shock). For the running procedure, a plastic barrier was placed between the shock grid and the treadmill line to prevent animals from resting on the top of the grid during the rehabilitation protocol. All mice were weighed every session, which consisted of 2 (short-term) or 12 (long-term) days of rehabilitation (Figure 2B). Finally, after every session, each animal received four small pieces of pasta (1.6 cm/piece) in their home-cages in order to balance the pasta ingestion between groups. After completing the rehabilitation treatments, mice were euthanized under deep anesthesia as described below to obtain brain, spleen, and plasma samples for further analysis.

### Grip strength test

The maximum forelimb force was assessed with the grip test as previously described (40). For the test, the mice were placed in a horizontal plane and when pulled backwards from the tail the animal exerts force in the grid. Scores of each animal were determined by averaging 6 trials. This test was performed before and after 24 h, 4 days, and 18 days of ischemia (corresponding 2 or 12 days of rehabilitation).

### Infarct volume assessment in animals

Animals were euthanized after 4 days of pMCAo (2 days after RHB) by transcardial perfusion of cold saline with deep anesthesia as described. Brains were removed and cut into 1 mm-thick coronal sections and stained with 2,5% of 2,3,4-triphenyl-2H-tetrazolium chloride (TTC; Sigma, MO, USA) for 10 min at room temperature (RT) when TTC solution was replaced by cold saline and images acquired for infarct quantification. Afterwards, ipsilateral and contralateral cortex were dissected for further molecular analyses, snap frozen in dry ice and stored at − 80°C until use. A treatment-blinded researcher measured the infarct volume by the ImageJ free software as described previously (39). Finally, the results were corrected for brain edema taking into account the following equation: infarct volume corrected = infarct volume/edema (ischemic area/contralateral area), and expressed in cubic millimeters (mm^3^).

### Glucose level monitoring

Glucose levels were monitored in a sub-group of animals before and after food restriction together with pMCAo (24 h and 4 days). Blood was obtained by making a small incision on the tail vein of mice, placing one droplet of blood on a glucose test strip and using a glucometer (GLUCOCARD^TM^ G+).

### Angiogenin measurements by ELISA

For human samples, serum levels of angiogenin were measured by ELISA kit (R&D systems, MN, USA). Samples from strokes were obtained before IRT (*n* = 18), after 1 month (*n* = 15), 3 months (*n* = 15), and 6 months (*n* = 15) of rehabilitation intervention, while 13 samples were tested for the non-ischemic control cohort (Figure 1). Briefly, 200 μl of diluted serum samples (1:200) were loaded to the sample wells to test angiogenin levels by ELISA following the manufacturer's instructions.

For mouse samples, venous blood was obtained from cardiac puncture under deep anesthesia and collected in EDTA tubes at day 2 or 12 post-rehabilitation and after sham surgery (Figure 2B). After centrifugation (3,000 g, 10 min at 4°C), plasma was separated and stored at − 80°C until analysis. Briefly, 100 μl of the diluted plasma (1:20) were loaded to the sample wells to quantify angiogenin levels by a sandwich ELISA kit (LSBio, WA, USA) according to manufacturer's instructions.

All samples were tested by duplicate and only values with a coefficient of variation < 20% were accepted for the statistical analysis. For those plates in which the inter-assay CV was higher than 20%, values were standardized prior to statistical analysis by calculating the Z-score value by substracting the mean and dividing by the standard deviation (*SD*) of each plate and adding three units to avoid results below zero in any sample.

### Flow cytometry for EPC cell counts in stroke and control cohorts

Fresh blood samples were collected in EDTA tubes from stroke patients before IRT, during the follow-up after 3–6 months of IRT and in control subjects as described.

Blood (4 ml) was diluted with 8 ml of PBS containing 2% of fetal bovine serum (Gibco, MD, USA) and the diluted blood was carefully placed over 5 ml of Ficoll-Paque Plus^TM^ (GE healthcare, Sweeden). Cells were centrifuged for 30 min (400 g) at RT. Mononuclear cell (MNC) layer was isolated and centrifuged for 5 min at RT (400 g) to discard the supernatant. Then, cell pellets were resuspended in 3 ml of PBA buffer (1% bovine serum albumin, 0.1% sodium azide in PBS) and 1 ml of blocking reagent was added (AB Human Serum, Invitrogen, CA, USA) and incubated for 10 min. Afterwards, 3 ml of the total mixture were used to incubate with primary antibodies and 1 ml was also incubated with corresponding fluorescent isotype control antibodies in order to control the non-specific antibody binding. Primary antibodies and isotypes were incubated for 45 min at RT [Antibodies: 0.3 μg/ml CD34-BV421, 0.3 μg/ml CD45-APC, and 0.75 μg/ml CD309-KDR-PE. Isotypes: 0.3 μg/ml IgG1-BV421, 0.75 μg/ml IgG1-PE (BD Pharmingen, CA, USA), and 0.3 μg/ml IGG1-APC (eBioscience, CA, USA)]. Tubes were centrifuged for 5 min at RT (350 g), discarding the supernatant, the cell pellet was resuspended in PBA buffer (0.8 ml for the sample and 0.5 ml for isotype) and transferred into 5 ml poliestirene plastic tubes. Before being analyzed, the samples were filtered through 30 μm Cell Tricks filters (Partec, Münster, Germany). Finally, 10^6^ events per sample and 1 to 2 × 10^4^ events for isotypes were analyzed using the BD LSRFortessa™ cell cytometer (BD Pharmingen, CA, USA). CD34^+^ and CD34^+^KDR^+^CD45^−^ populations were analyzed using FCS Express™ version 3, Research Edition (DeNovo Software, CA, USA). Extreme values represented in box-plots were excluded from the analyses.

### EPCS cultures from mouse spleens

Mouse spleens were used to obtain early EPCs as previously described (41). Spleens were obtained after 2 or 12 days of RHB treatment and two spleens from the same group were pooled for cultures. Spleens were mechanically minced, placed at 37°C for 10 min in a 1 mM EDTA solution and run through a 40-μm nylon membrane to obtain a cell suspension. Mononuclear cells (MNCs) were obtained by density gradient centrifugation (400 g, 20 min) with Ficoll-Paque Plus (GE healthcare, Sweeden), shortly washed with red blood cells lysis solution (150 mmol/L NH_4_Cl, 10 mmol/L NaHCO_3_ and 0.1 mmol/L EDTA in distilled water) and gently washed with complete endothelial growth medium-2 (EGM-2; Clonetics®, CA, USA), which is composed of endothelial basal medium (EBM) containing 20% fetal bovine serum (FBS), human epidermal growth factor (hEGF), vascular endothelial growth factor (VEGF), human basic fibroblast growth factor (hFGF-B), insulin like growth factor 1 (R3-IGF-1), GA-1000 (gentamicin and amphoterecin-B), heparin, hydrocortisone, and ascorbic acid. Isolated MNCs were finally resuspended in EGM-2 and 10^7^ MNCs were seeded on fibronectin-coated 12-well cell culture plates and incubated in 5% CO_2_ at 37°C. Under daily observation, first media change was performed 3 days after plating and, thereafter, media was changed every 2 days. At day 5, images from four representatives fields/well were taken at 100X magnification (IX71, Olympus). Before data analysis, extreme values were excluded by mean ± 2 SD criteria.

### Immunohistochemistry (IHC)

IHC was performed in human and mouse brain slices for angiogenin, NeuN antibodies, and lectin blood-vessel marker. For human tissue, post-mortem brain samples from stroke patients (see Supplementary Table 1 for details) were collected from infarct tissue and healthy contralateral hemispheres by an experienced neuropathologist and supported by computed tomography images. For animal samples, mice under 12 days of rehabilitative treatment received daily intraperitoneal injections of 5-Bromo-2′-deoxyuridine (BrdU, 50mg/kg in saline, B9285, Sigma-Aldrich, MO, USA) (42), which started after 48 h of pMCAo. Later Dylight 594-labeled tomato lectin (80 μg/mouse, DL-1177, Vector Laboratories, USA) was injected intravenously (retro-orbitally) 10 min before euthanasia. Afterwards, animals were euthanized by transcardial perfusion with cold paraformaldehyde (PFA, 4%) under deep anesthesia. Both human and animal brains were fixed using 4% paraformaldehyde overnight or 2 h, respectively, changed to 30% sucrose until the brains sank, embedded in OCT and kept frozen at −80°C until use. Brain sections were obtained by cutting 12 μm-thickness slices in a cryostat. Human and mouse slices were tempered for 30 min and human sections were fixed with cold acetone for 15 min. Tissue slices were washed three times: 5 min at RT with 0.1% PBS-Tween, 5 min with 0.3%-PBS-Triton X-100, and 5 min with 0.1% PBS-Tween. Samples were blocked by using 0.1% PBS-Tween containing 1% of BSA (Sigma-Aldrich, MO, USA) and 5% of goat serum (Merck Millipore, MA, USA) for 1 h. Then, slices were incubated overnight with the following primary antibodies: 1:100 Angiogenin (NBP2-41185; Novus, Littleton CO), 1:100 NeuN (MAB377X; Merck Millipore, Billerica, MA), and for human vessel analysis 1:100 lectin glycoprotein (RL-1062; Vector labs. CA, USA) was used. Before the secondary antibody incubation, sections were washed three times for 5 min at RT with 0.1% PBS-Tween. As secondary antibodies, Alexa fluor 488 goat anti-rabbit IgG (1:1,000, mouse; 1:500, human) and Alexa fluor 568 goat anti-mouse IgG (1:500) (Invitrogen, CA, USA) were incubated for 1 h at RT. Later, three washes for 5 min with 0.1% PBS-Tween at RT were performed. Additionally, human samples were incubated 10 min at RT with Sudan Black B staining to reduce the brain tissue autofluorescence and washed with 0.1% PBS-Tween three times for 5 min after the incubation. Human and mice sections were then mounted in Vectashield^TM^ with DAPI (H-1200, Vector Laboratories, USA) to stain the cell nuclei and analyzed with Olympus BX61 microscope (Olympus, Japan). Finally, two slices of infarct and healthy contralateral areas from three stroke patients were double-stained for NeuN/angiogenin and lectin/angiogenin and the total angiogenin positive area was calculated using ImageJ free software.

For mice sections analysis, two slices from six animals were imaged at 100X magnification. Two images from each section were captured from the infarct boundary area and contralateral cortex. Total area of angiogenin positive staining was calculated in mice sections using ImageJ free software to compare ischemic side vs. contralateral side. Image analysis was performed by a blinded investigator.

### Western blot

Western blot analysis for angiogenin was performed in human and mouse ischemic and non-ischemic brain cortex homogenates. Human stroke brain homogenates (see Supplementary Table 1 for details), mice ischemic brain homogenates (core of infarct, 24 h post-pMCAo) and corresponding contralateral tissues were homogenized with freshly prepared ice-cold lysis buffer containing 50 mM Tris-HCl, 150 mM NaCl, 5 mM CaCl_2_, 0.05% BRIJ-35, 0.02% NaN_3_, 1% Triton X-100, 1% phenylmethanesulfonyl fluoride (PMSF; Sigma-Aldrich, Switzerland), and 0.5% aprotinin (Sigma-Aldrich, USA). Lysates were collected from the supernatant after centrifugation at 15,300 g for 12 min at 4°C and Pierce^TM^ Comassie (Bradford) assay was used to determine the total protein content (Thermo Scientific^TM^, IL, USA). A total amount of 40 μg for human samples and 10 μg for mouse samples was diluted in Laemmli Buffer (5%, 2-mercaptoethanol), incubated 5 min at 95°C and run into 12% polyacrylamide gel electrophoresis, transferred into PVDF membranes (Thermo Scientific^TM^, IL, USA), blocked for 1 h with 10% non-fat milk [PBS, 0.1% Tween 20 (Sigma-Aldrich, MO, USA)] and incubated overnight at 4°C on a shaker with anti-angiogenin (1:200, NBP2-41185, Novus, CO, USA) or β-actin (1:5000, A5316, Sigma-Aldrich, MO, USA). The membrane was then washed three times (PBS-0.1% Tween) and incubated with secondary antibodies (GE Healthcare, UK) anti-rabbit-horseradish peroxidase from donkey or anti-mouse horseradish peroxidase from sheep (1:2,000, respectively) for 1 h at RT with gentle agitation. Finally, membranes were washed three times (PBS-0.1%Tween) and incubated with Pierce® ECL Western Blotting Substrate (Thermo Scientific^TM^, IL, USA) and signal visualized with Fujifilm FPM-100A films. Scanned western blots were quantified using the ImageJ free software. Results are expressed in arbitrary units and angiogenin band-intensities were corrected by actin band-intensities. Molecular weight markers were also run for reference values.

### Quantitative reverse transcriptase pcr (QRT-PCR) for angiogenin expression

Mice tissues from 2 and 12 days of rehabilitation were used for angiogenin RNA isolation with the PARIS ^TM^ kit (Invitrogen, CA, USA), following the manufacturer's procedure. The quality and quantity of RNA was measured using Nanodrop Spectrophotometer and cDNA was synthesized using a High-Capacity cDNA Reverse Transcription Kit (Applied Biosystems, Foster City, CA, USA). RT-PCR reaction was performed using a mixture of 5 μL of TaqMan® Universal PCR Master Mix (Applied Biosystems, Foster City, CA, USA), 0.5 μL of TaqMan® Gene Expression Assay (Gapdh: Mm99999915_g1, angiogenin (Ang): Mm01316661_m1; Applied Biosystems, Foster City, CA, USA), 3.5 μL of RNase-free water and 1 μL of cDNA sample. A sample calibrator was used to compare samples between different assay plates. Samples were run in triplicate using 384-well plates on 7900HT Fast Real-Time PCR System (Applied Biosystems, Foster City, CA, USA). Gene expression results were expressed in RQ (relative quantification).

### Statistical analysis

The SPSS 20.0 package was used for statistical analyses. Descriptive statistics were used to define demographic data and risk factors, stroke characteristics, functional scores, angiogenin, and cell analysis results. Chi-square tests were conducted for categorical variables. The normality of continuous variables was assessed by the Shapiro-Wilk test (*N* < 30) and Kolmogorov-Smirnov (*N* ≥ 30). Normally distributed variables were analyzed by the ANOVA and LSD *post-hoc* or *t*-test, while Mann-Whitney U-test or Kruskal Wallis test were used for non-normally distributed variables. For the analysis of repeated measures, paired *t*-tests or repeated measures ANOVA followed by the Bonferroni post hoc test were performed for normally distributed variables, while the Friedman followed by Wilcoxon tests were used for non-normal distributions. Bar graphs represent mean ± SEM or IQR according to normal or non-normal distribution. For correlation analysis, Pearson (normal distribution) or Spearman (non-normal distribution) tests were used. Results with a *p* < 0.05 were considered statistically significant. Statistical trends (*p* < 0.1) are also reported in the results section.

## Results

### Characteristics of the study cohorts

The baseline characteristics of the stroke rehabilitation cohort and control subjects are described in Table 1. In summary, non-stroke controls were significantly older (64 vs. 54.06 years, *p* = 0.002), had higher alcohol intake (*p* = 0.034), had a higher body mass index (*p* = 0.019) and were more often on antihypertensive drugs (*p* = 0.04) than stroke patients.

**Table 1:** Baseline characteristics of the control and Stroke cohorts.

	**Control cohort (*n* = 18)**	**Stroke cohort (*n* = 18)**	***P*-value**
Age	64 ± 7.7	54 ± 9.6	0.002*
Sex (male)	50 (9)	72.2 (13)	0.171
**RISK FACTORS**			
Alcohol	50 (9)	16.7 (3)	0.034*
Tobacco	16.7 (3)	33.3 (6)	0.443
Hypertension	83.3 (15)	66.7 (12)	0.443
Dyslipidemia	66.7 (12)	44.4 (8)	0.18
Diabetes mellitus	22.2 (4)	16.7 (3)	1
Atrial fibrillation	0 (0)	11.1 (2)	0.486
Obesity	66.7 (12)	38.9 (7)	0.095
Body mass index (kg/m^2^)	26 ± 3.3	23.2 ± 3.5	0.019*
**COMORBIDITIES**			
Osteoarticular disorders	22.2 (4)	11.1 (2)	0.658
Ischemic cardiopathy	0 (0)	5.6 (1)	1
Psychiatric disorders	22.2 (4)	22.2 (4)	1
**PREVIOUS EXERCISE**			
Physical activity	77.8 (14)	47.1 (8)	0.06
Physical activity (h)	7 (5–10)	0 (0–7)	0.116
**PREVIOUS MEDICATION**			
Anti-platelets	27.8 (5)	16.7 (3)	0.674
Anti-coagulants	0 (0)	5.6 (1)	1
Statins	38.9 (7)	27.8 (5)	1
Anti-hypertensives	77.8 (14)	44.4 (8)	0.040*
Anti-diabetic	16.7 (3)	11.1 (2)	0.639

Variables are expressed as percentage, mean ± SD, median (IQR), and the number of cases as (n); h, hours.

* *p-value < 0.05*.

Table 2 shows the clinical features associated with the ischemic stroke. In summary, the median National Institutes of Health Stroke Scale (NIHSS) score on admission was 8 (5–10), and after 3–4 days, it improved to 5 (4–9) with 38.9% of patients showing a neurological improvement (≥4 points). Strokes were mainly located in the carotid territory (77.8%), presenting large infarcts (TACI, 55.6%), with more than half affecting the left hemisphere (55.6%). Blood samples were taken at 11.5 days after the ischemic event, and the mean number of days for patients to enroll in IRT was 12.4 days.

**Table 2:** Clinical characteristics of the Stroke cohort.

	**Stroke cohort (*n* = 18)**
NIHSS score at admission	8 (5–10)
NHISS motor score at admission	4 (2–7)
NHISS score after 3–4 d	5 (4–9)
NHISS motor score after 3–4 d	2.5 (2–5)
**EARLY NEUROLOGICAL OUTCOMES**	
Improvement	38.9 (7)
Stability	50 (9)
Worsening	11.1 (2)
**ETIOLOGY**	
Lacunar	11.1 (2)
Cardioembolic	33.3 (6)
Atherothrombotic	11.1 (2)
Others	16.7 (3)
Unknown	27.8 (5)
**LOCATION, TERRITORY**	
Vertebrobasilar	22.2 (4)
Carotid	77.8 (14)
**STROKE LATERALITY**	
Right	44.4 (8)
Left	55.6 (10)
**OCSP CLASSIFICATION**	
TACI	55.6 (10)
LACI	16.7 (3)
PACI	11.1 (2)
POCI	16.7 (3)
**OTHER**	
Thrombolytic therapy	35.3 (6)
Hemorrhagic transformation	17.6 (3)
Time stroke-baseline sample before IRT (d)	11.5 ± 5.7
Time stroke-evaluation before IRT (d)	12.4 ± 5

*Variables are expressed as percentage, mean ± SD, or median (interquartile range). Early neurological outcomes were defined as improvement (decrease ≥4 points in the NHISS admission score), worsening (increase ≥4 in the NHISS admission score), or stability (as any other change in the NHISS admission score)*.

*OCSP, Oxfordshire Community Stroke Project; TACI, total anterior cerebral infarct; LACI, lacunar cerebral infarct; PACI, partial anterior cerebral infarct; POCI, posterior cerebral infarct; IRT, intensive rehabilitation therapy; d, days*.

### Angiogenin, CD34^+^, and CD34^+^/KDR^+^/CD45^−^ cells increase after stroke related to IRT

**Figure 3:**
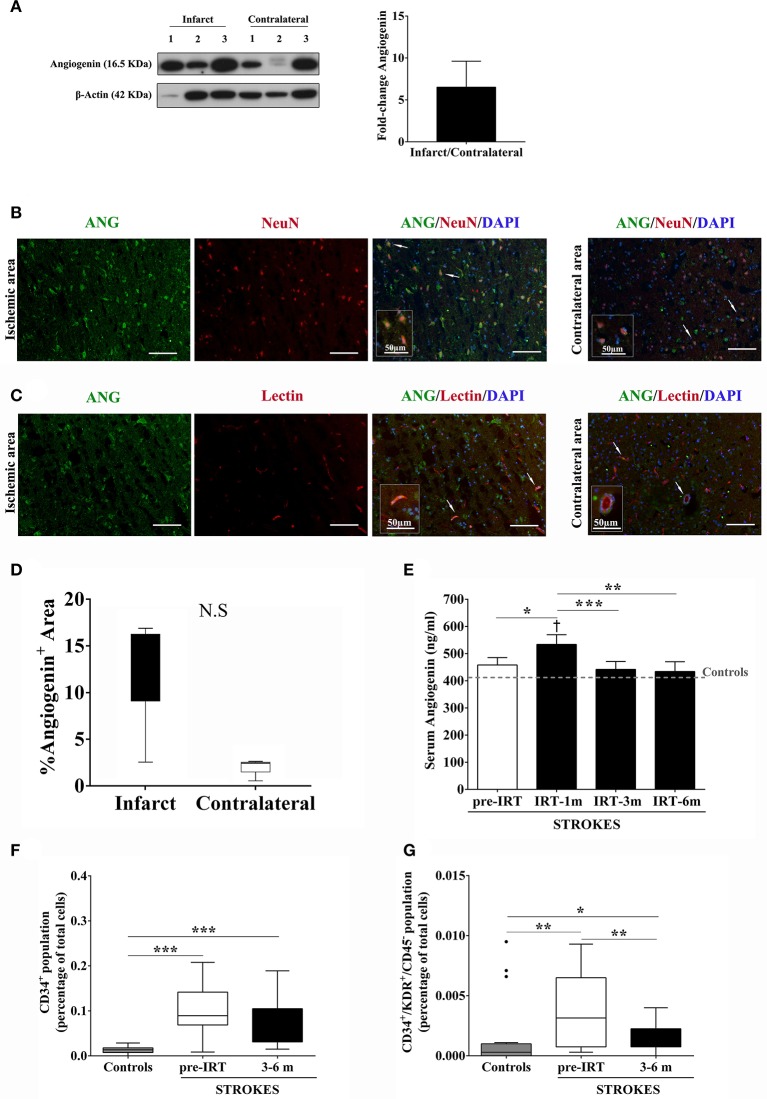
Angiogenin and EPCs modulation after human stroke and rehabilitation. **(A)** Infarct tissue and contralateral brain homogenates (≤ 4 days) were analyzed by western blot for angiogenin (*n* = 3). The densitometry results (arbitrary units) were corrected by the actin load and bar graph showing the angiogenin fold-change of the ipsilateral vs. contralateral signal. **(B**,**C)** Representative immunofluorescence images of ischemic and contralateral areas, respectively, of stroke patients showing angiogenin^+^/NeuN^+^ cortical neurons and angiogenin^+^/lectin^+^ vessels**. (D)** Box plots representing the percentage of the fluorescent Angiogenin^+^ area in infarcted and non-infarcted brain samples from stroke patients (*n* = 3). **(E)** Bar graphs showing the temporal profile of serum angiogenin levels during IRT. *p < 0.05; **p < 0.01, and ***p < 0.001; *p* < 0.05 vs. Controls (Controls *n* = 13; Strokes in IRT *n* = 15–18). **(F,G)** Box plots representing cell populations with angiogenic potential such as CD34^+^ and CD34^+^/KDR^+^/CD45^−^ cell populations; *p < 0.05, **p < 0.01, ***p < 0.001; Controls *n* = 17 and Strokes in IRT *n* = 15–17. Bar graphs are represented as the mean ± *SEM*, and box plots are represented as the median (IQR). NS, Non-significant; IRT, intensive rehabilitation therapy; m, month.

More brain angiogenin content was found during the acute phase of stroke in infarct tissue compared to the contralateral hemisphere (mean fold increase of 6.5; Figure 3A and Supplementary Figure 1), although differences were not significant. Immunohistochemistry experiments revealed that this expression of brain angiogenin was mainly localized in neurons (NeuN^+^, Figure 3B) and in vessel-like structures (lectin^+^, Figure 3C), with higher expression in infarct tissue (Figure 3D).

In the context of post-stroke rehabilitation, circulating angiogenin levels were significantly increased after 1 month of IRT compared to controls (*p* = 0.012) and compared to pre-rehabilitation values (*p* = 0.036), decreasing after 3 months (*p* = 0.001) and 6 months (*p* = 0.002) of IRT as shown in Figure 3E. Regarding EPCs populations, we observed that CD34^+^ and CD34^+^/KDR^+^/CD45^−^ cells increased after stroke compared to controls (*p* < 0.001 and *p* = 0.005, respectively) and were elevated during the follow-up (3–6 months) compared to controls (*p* < 0.001 and *p* = 0.028, respectively; Figures 3F,**G**) despite a decrease in CD34^+^/KDR^+^/CD45^−^ cells compared to pre-IRT levels (*p* = 0.004; Figure 3G). No association was observed between angiogenin and EPCs at baseline, although serum angiogenin at 1 and 3 months associated negatively with CD34^+^ cells during the follow up (*R* = −0.636, *p* = 0.011; *R* = −0.679, *p* = 0.005; respectively).

### High angiogenin during IRT is associated with better neurological outcome

**Figure 4:**
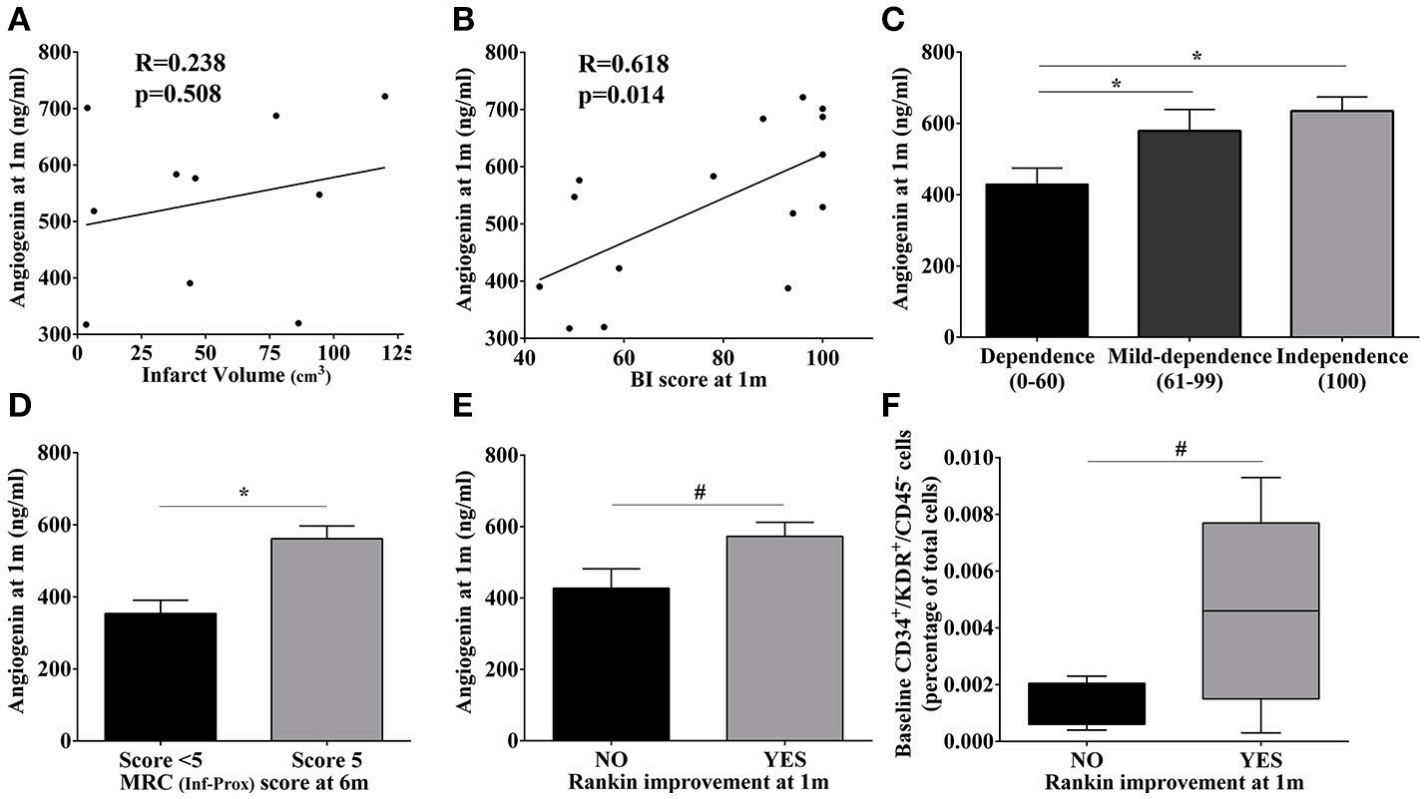
Association of molecular and cellular markers with infarct volume and motor/functional scores in stroke patients. **(A**,**B)** Scatter plot showing the correlations of angiogenin levels at 1 month with infarct volume and the BI score, respectively. **(C–E)** Bar graphs showing angiogenin levels at 1 month with BI score categories, MRC scores, and Rankin improvement at different time points;*p < 0.05, ^#^*p* < 0.1. **(F)** Bar graphs representing baseline CD34^+^/KDR^+^/CD45^−^ cells according to the Rankin improvement at 1 month. Bar graphs are represented as the mean ± SEM, and box plots are represented as the median (IQR). BI, Barthel index; m, month; MRC, Medical Research Council; Inf-Prox, Inferior-proximal.

Neither baseline angiogenin nor EPC populations were associated with the baseline NIHSS score or infarct volume (*p* > 0.05; data not shown). Additionally, the increased angiogenin levels observed at 1 month were not associated with baseline infarct volume (*p* > 0.05; Figure 4A). Regarding improvement, a strong correlation was found between angiogenin levels and Barthel Index scores at 1 month (*R* = 0.618, *p* = 0.014), and patients with higher Barthel Index scores (mild and complete independence) presented the highest angiogenin levels (*p* = 0.048 and *p* = 0.015, vs. severe dependence); see Figures 4B,C. Similarly, patients presenting the highest angiogenin levels presented a normal MRC score at 6 months (*p* = 0.043; Figure 4D) and functional outcomes assessed by the Rankin scale also showed a trend toward better improvement at 1 month (*p* = 0.07; Figure 4E), showing the association between high angiogenin levels and better neurological improvements.

Regarding circulating EPC populations, we observed a trend only toward higher CD34^+^ levels during IRT follow-up and normal MRC scores for superior-proximal and distal extremities at 3 months (*p* = 0.086, respectively, data not shown), whereas the highest baseline levels of CD34^+^/KDR^+^/CD45^−^ cells were observed in those patients presenting a Rankin improvement at 1 month (*p* = 0.09; Figure 4F).

### Characteristics of the mouse rehabilitation models after cerebral ischemia

**Figure 5:**
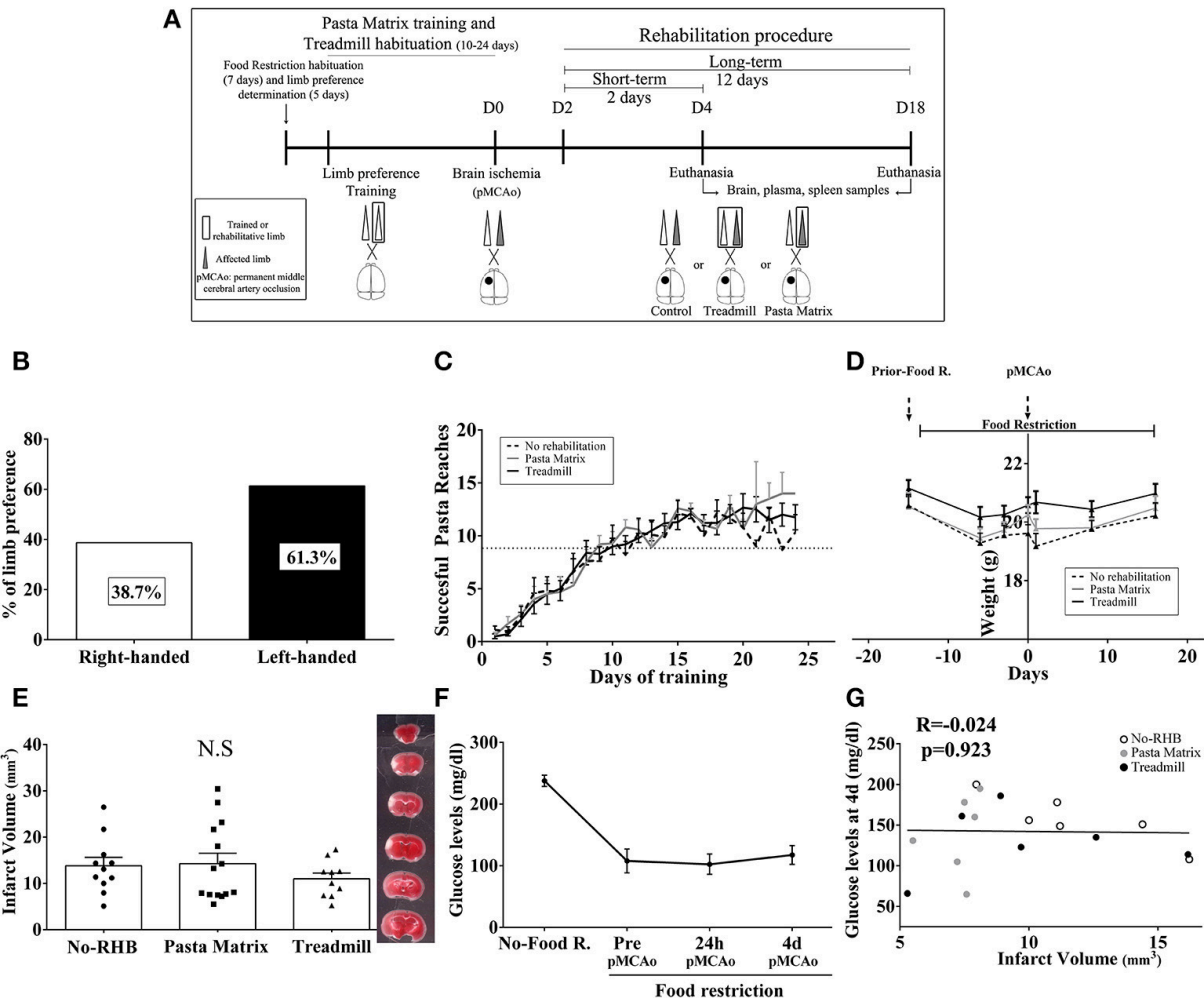
**(A)** Mouse rehabilitation model. **(B)** Graph showing limb laterality preference in our cohort of mice (*n* = 74). **(C)** Graph representing the temporal profile of pasta matrix reaching-task training in *n* = 10 animals/group. **(D)** Body-weight graph during the entire procedure in *n* = 10 animals/group. **(E)** Bar graphs showing the mean infarct volume during the short-term phase of rehabilitation with no significant differences between groups (*n* = 10–14). **(F)** The glucose monitoring graph during acute cerebral ischemia **(F)**. **(G)** Scatterplot showing no association between infarct volume and glucose levels during the short-term phase of rehabilitation (4 days pMCAo, *n* = 6/group). Data are represented as percentage, mean ± SEM, or single values in the scatter plot. NS, Non-significant; RHB, rehabilitation; pMCAo, permanent middle cerebral artery occlusion; R, restriction; d, days.

Our cohort of mice (Figure 5A) presented a preference for the left forelimb (61.3%) vs. right forelimb (38.7%) (Figure 5B) and learned the pasta matrix-reaching task as expected between day 10 and 24 of training (Figure 5C). As a consequence of food restriction, the weights did not increase, although no significant weight loss was observed and no significant differences were observed between rehabilitation groups (Figure 5D).

Importantly, at the beginning of rehabilitation (2 days), infarct volumes were similar among groups (*p* > 0.05; Figure 5E). Food restriction decreased the levels of blood glucose as expected, but no correlations with infarct size were observed (*p* > 0.05; Figures 5F,G).

### Angiogenin and endothelial progenitor cells are modulated in the rehabilitation models after cerebral ischemia

**Figure 6:**
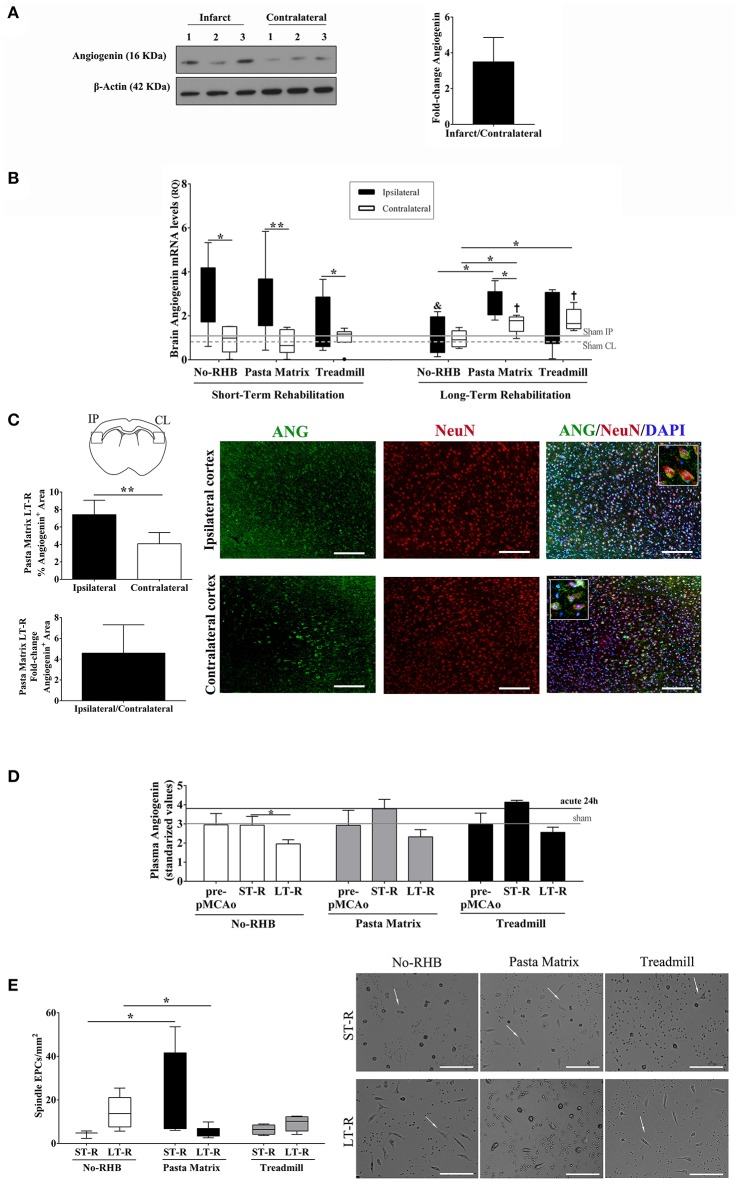
Angiogenin and EPCs modulation after mouse cerebral ischemia and rehabilitation. **(A)** Infarct and contralateral mouse brain cortical homogenates were analyzed by western blot to quantify angiogenin protein (*n* = 3). The densitometry results (arbitrary units) were corrected by the actin load and are represented as fold-change of the ipsilateral vs. contralateral signal. **(B)** Bar graphs representing the angiogenin RNA expression in the ischemic and contralateral cortex in the short-term (*n* = 6–9) and long-term rehabilitation groups (*n* = 5), **p < 0.01, *p < 0.05 as indicated by horizontal lines. Non-RHB ipsilateral short-term vs. long-term; ^&^*p* < 0.05. Pasta Matrix and Treadmill contralateral short-term vs. long-term; †*p* < 0.05. **(C)** Bar graphs showing the percentage of the Angiogenin^+^ area in the pasta matrix long-term rehabilitation group (*n* = 6) together with images of representative brains with inserts showing co-localization of angiogenin in neurons: scale bar represents 100 μm. *p < 0.05. **(D)** Graph showing the plasma angiogenin temporal profile of No-RHB, Pasta matrix, and Treadmill groups (*n* = 3–4/group); *p < 0.05. **(E)** Box plots representing the cell density of EPCs from the three different RHB groups (*n* = 3–5 short-term; *n* = 6–7 long-term) and representative images of the primary cultures; scale bar represents 250 μm. Data are represented as the mean ± SEM or as box plots indicating the median (IQR). IP, ipsilateral; CL, contralateral; ST-R, short-term rehabilitation; LT-R, long-term rehabilitation; No-RHB, No-Rehabilitation; EPC, endothelial progenitor cells.

Angiogenin was expressed in brain tissue homogenates 24 h after ischemia before rehabilitation, presenting a non-significant 3.5-fold increase in the ipsilateral vs. the contralateral hemisphere (Figure 6A and Supplementary Figure 2).

RNA expression of angiogenin increased early after rehabilitation in the ipsilateral hemisphere in all groups (*p* < 0.05), but angiogenin did not correlate with the infarct volume (data not shown). Importantly, angiogenin remained elevated in the ipsilateral hemisphere only in mice performing the pasta-matrix-reaching task for 12 days compared to the contralateral hemisphere (*p* = 0.043) and compared to the ipsilateral of non-RHB mice (*p* = 0.016). The contralateral hemispheres from the pasta matrix and treadmill groups also showed higher levels of angiogenin than the contralateral of non-RHB mice (*p* = 0.028 and *p* = 0.016, respectively); see Figure 6B. We observed a decrease in ipsilateral angiogenin over time only in non-RHB mice (*p* = 0.045), whereas ipsilateral angiogenin from the pasta matrix and treadmill groups remained elevated over time, and contralateral angiogenin increased in both rehabilitation groups (*p* = 0.014 and *p* = 0.007, respectively).

We validated the RNA findings in the pasta matrix reaching-task model at the protein level by IHC, confirming that angiogenin was increased in the ipsilateral vs. the contralateral hemisphere after 12 days of rehabilitation (*p* = 0.003), with a mean fold increase of 4.6, as shown in Figure 6C. Strong angiogenin expression was identified in neurons of the ipsilateral cortex (see details in Figure 6C).

The analysis of blood angiogenin in the mouse rehabilitation models showed a decrease at 12 days after rehabilitation only in the control group (*p* = 0.029 vs. short-term rehabilitation), while the pasta matrix and treadmill presented an increase at the beginning of rehabilitation that remained elevated at the end of the treatment; however, these differences were not significant (Figure 6D).

Forelimb force was decreased 24 h after ischemia (*p* < 0.001; data not shown); however, there were no differences between groups after rehabilitation.

Finally, spleen-derived EPCs from the pasta matrix reaching-task rehabilitation group showed an increase in the number of spindle-shaped cells compared to the non-rehabilitation in the short-term (*p* = 0.025). In the long-term, the number of cells decreased compared to that in the non-rehabilitation group (*p* = 0.025); see Figure 6E.

## Discussion

Currently, the only approved treatment for recovery after the acute phase of stroke is rehabilitation with the aim of maximizing independence and improving physical disabilities. In this study, we have examined the role of angiogenin and EPCs during IRT in ischemic stroke patients and in a neurorehabilitation animal model after cerebral ischemia. Our results describe for the first time an increase of angiogenin and EPCs levels during rehabilitation therapy after stroke. We also identify an association between serum angiogenin levels and functional and motor improvement of outcome measurements. These observations are supported by *in vivo* experiments in a neurorehabilitation animal model where RNA expression and primary EPCs are increased after rehabilitation interventions. These data suggest that angiogenin and EPCs are modulated during rehabilitation therapies and might act as biomarkers to monitor patient status or to potentially serve as therapeutic targets.

Despite the enormous therapeutic advances with reperfusion therapies in combination with well-coordinated teams in stroke units, one-third of stroke patients still present functional deficits (43, 44). With this scenario, the only therapeutic opportunities are neurorehabilitation programs that are evidence-based multidisciplinary interventions to restore the impaired function and improve patient independence and quality of life (5, 45). However, the patient response to rehabilitation is heterogeneous and might depend on individual endogenous neurorepair mechanisms (46), thus limiting recovery during rehabilitation. In this regard, preclinical and clinical studies should aim to understand the ongoing mechanisms during rehabilitation and to identify new biomarkers to monitor patient baseline conditions, monitor the neurorepair process, and adjust personal rehabilitation programs to maximize the optimal recovery.

Angiogenin is a member of the ribonuclease superfamily and is expressed in many cell types. Angiogenin acts as a potent angiogenic factor that can trigger a wide range of biological processes, such as proliferation, cell migration, invasion, and formation of tubular structures (47). However, uncontrolled activity of angiogenin is implicated in pathological processes. A high expression of angiogenin has been described in different types of cancer (48), and mutations in the angiogenin gene have been characterized in amyotrophic lateral sclerosis (49) and Parkinson's disease (50). Angiogenin has also been studied in cardiovascular diseases showing an increase in blood in chronic heart failure or in acute coronary syndrome in association with severity or prognosis (51, 52). In the context of stroke, Huang et al. reported that higher serum angiogenin levels within the first week after stroke are associated with larger infarct size (53). This up-regulation of angiogenin in the acute phase of cardiovascular diseases has also been observed in our study by identifying angiogenin in neurons or vessels of the brain within the first 4 days in infarcted areas. In this regard, several authors have used cell culture experiments to reveal that angiogenin is expressed in motoneurons and is protective under hypoxic conditions (49). But angiogenin also mediates tumor angiogenesis in cancer (54).

In the present study, we have described the temporal profile of angiogenin from the subacute phase to the most chronic phases after ischemic stroke during rehabilitation. In patients, before starting IRT, angiogenin levels were not different from those of the controls. This result is in accordance with a previous publication showing a decrease in angiogenin levels at day 14 after an initial acute increase after 48 h (53). In our study, angiogenin levels measured before IRT were not correlated with infarct size, probably because those measures were performed after the most acute phase of the disease. One of the most interesting findings in the present study is the increase in blood angiogenin at 1 month after starting IRT, followed by a decrease at 3 and 6 months. More importantly, this increase is clearly associated with an improvement in Barthel Index, MRC, and Rankin scores, confirming the potential use of angiogenin as a biomarker to monitor rehabilitation.

In parallel, the pasta matrix and treadmill exercise for rodents were used to support our findings in stroke patients. The pasta matrix task has recently been included as a rehabilitative approach in mice by Kerr et al. in 2014 (38) as an experimental task-oriented test to study the brain mechanisms targeting only the affected or the contralateral forelimb. The pasta-matrix approach requires a food-restricted diet, which was established for all animals in the study and caused an expected hypoglycemic status. Importantly, the achieved glucose levels during ischemia and rehabilitation were not different between groups, and there was no association with infarct size. The treadmill task has been widely used to study exercise-related mechanisms after ischemia in rodents (20, 55), with several authors reporting neuroprotective effects when the activity is performed before ischemia or within 24 h of ischemia (17, 20, 55). As observed in human stroke samples, angiogenin was found in the mouse brain after cerebral ischemia and was increased in the ipsilateral cortex and more specifically in neurons. Another study conducted in a rat stroke model previously described elevated brain angiogenin levels during the first week with a clear nuclear expression in neurons (22).

We have shown an increase in ipsilateral angiogenin RNA expression in all groups at the beginning of rehabilitation with no differences between groups; importantly, higher RNA expression was found after 12 days of rehabilitation in the task-oriented group compared to the non-rehabilitation group. Additionally, the contralateral brains of both rehabilitation groups exhibited increased angiogenin levels compared to the non-rehabilitation group. These observations link brain angiogenin with rehabilitation tasks, being different in task-oriented exercises for the impaired forelimb than in general-running exercise where both extremities are used. Recently, Zhang et al. have recently reported that a skill reaching training (task-oriented) enhanced more axonal plasticity and motor recovery than forced exercise via running wheels (56), suggesting a different activated-routes between both rehabilitation strategies. This could be linked to our finding of enhanced angiogenin RNA expression in cortical neurons of the ipsilateral hemisphere of the pasta matrix reaching-task group, validated at protein level mainly in cortical neurons.

Unfortunately, we could not demonstrate a functional improvement on the grip strength meter test in the mice with overexpressed brain angiogenin that received rehabilitation. We attribute this result to the spontaneous recovery observed in mice (40) and the need to evaluate the forelimb force separately between affected and non-affected sides.

Finally, EPCs have been investigated as key elements of neovascularization and for their use as biomarkers after stroke during rehabilitation. These cells have the capability to form new vessels in the adult, can mobilize to specific areas in response to ischemia and differentiate to endothelial cells (24, 26, 41). Different studies in stroke patients have shown an increase in circulating EPCs in the acute and subacute phases, and several studies have reported a peak in EPCs after 7 days of ischemic stroke associated with better outcome (27) followed by a decrease in the EPCs counts at 3 months (57). In our study, we report that the initial stroke-driven increase in EPCs is maintained during rehabilitation follow-up after several months. Finally, our pre-clinical experiments have revealed an early increase in spleen-derived EPCs in culture early after pasta-matrix rehabilitation but not in long-term. According to previous studies, we were expecting to also observe an increase in the mice undergoing daily exercise (58). Some differences could be explained by the fact that we have established a model of forced vs. voluntary exercise (58) or that our food restriction conditions could influence the spleen as described by others (59).

In conclusion, we have described for the first time the modulation of angiogenin and EPCs in stroke patients under IRT and in a mouse model of post-stroke rehabilitation task-oriented exercise. Our findings suggest that these molecules may be novel biomarkers to monitor and predict stroke recovery.

It is important to remark that the main limitation of our study is that we have not included a control cohort of stroke patients of similar characteristics who did not receive IRT to truly elucidate the role of rehabilitation in the modulation of angiogenin and EPCs. Ethical principles demand that all patients admitted to our hospital who are candidates for receiving IRT must be enrolled in the program. Future multicentric studies including other rehabilitation programs, and enlarging the number of studied patients should be designed in the future. As an approach to this non-rehabilitation condition after stroke, we have presented a pre-clinical model of stroke with or without rehabilitation intervention. The multicentric approach should be also considered for future pre-clinical studies including different occlusion models and other rehabilitation models.

## Author contributions

MG-S and AR conceived and designed the experiments; MG-S, AR, XB, NG, and SR organized the database and contributed to data collection; MG-S and DG performed the statistical analysis; MG-S, AM, GC, AF, and AB participated in data acquisition. MG-S and AR wrote the first draft of the manuscript. JM contributed to review the manuscript. All authors read and approved the final manuscript.

### Conflict of interest statement

The authors declare that the research was conducted in the absence of any commercial or financial relationships that could be construed as a potential conflict of interest.
