# Supplementary material for: Importance of Angiogenin and Endothelial Progenitor Cells After Rehabilitation Both in Ischemic Stroke Patients and in a Mouse Model of Cerebral Ischemia
Source: Front Neurol. 2018 Jun 29;9:508. doi: 10.3389/fneur.2018.00508 (PMC6034071; doi:10.3389/fneur.2018.00508)

SUPPLEMENTARY MATERIAL

Importance of Angiogenin and Endothelial Progenitor Cells after Rehabilitation both in Ischemic Stroke Patients and in a Mouse Model of Cerebral Ischemia.

Marina Gabriel-Salazar[a], Anna Morancho[a], Susana Rodriguez[b], Xavi Buxó[b], Nicolás García[b], Guillem Colell[a], Albert Fernandez[a], Dolors Giralt[a], Alejandro Bustamante[a], Joan Montaner[a], Anna Rosell[a]*.

[a]Neurovascular Research Laboratory and Neurology Department, Vall d'Hebron Research Institute, Universitat Autònoma de Barcelona, Barcelona, Spain

[b]Unidad de Rehabilitación Neurológica y Daño Cerebral, Hospital Vall d'Hebron, Barcelona, Spain.

Supplementary Table 1: Demographic and clinical data from postmortem samples of ischemic stroke patients.

Sex	Age	T-O-D (h)	PMI (h)	IHC	WB
Female	83	100	14.5		X
Male	84	40	8	X	X
Female	73	44	4	X	
Male	75	19	5	X	X

Abbreviations: T-O-D: time from onset of stroke symptoms to death; h:hours; PMI: postmortem interval (from death to brain samples collection) ;IHC: immunohistochemistry; WB: western blot.

Supplementary Figure 1: Full image of western blot shown in Fig.3A.

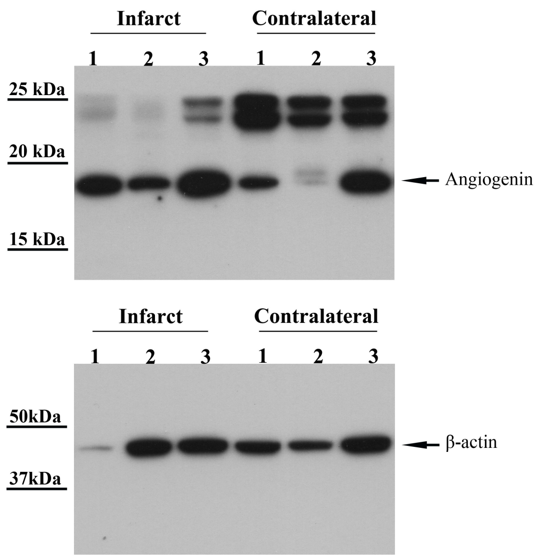

Supplementary Figure 2: Full image of western blot shown in Fig.6A.

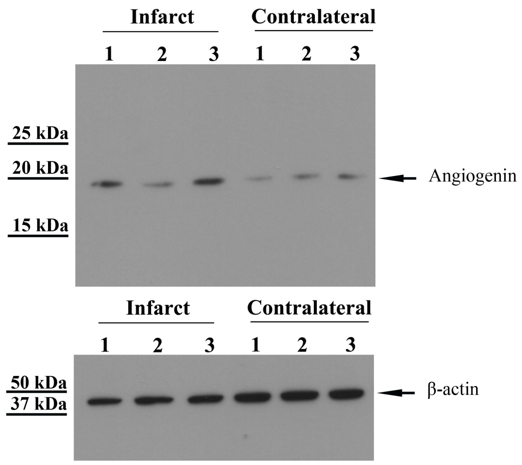

Supplement: Supplementary file 1 [file Presentation_1.pdf]
