# Supplementary figures and images for: Importance of Angiogenin and Endothelial Progenitor Cells After Rehabilitation Both in Ischemic Stroke Patients and in a Mouse Model of Cerebral Ischemia
Source: Front Neurol. 2018 Jun 29;9:508. doi: 10.3389/fneur.2018.00508 (PMC6034071; doi:10.3389/fneur.2018.00508)

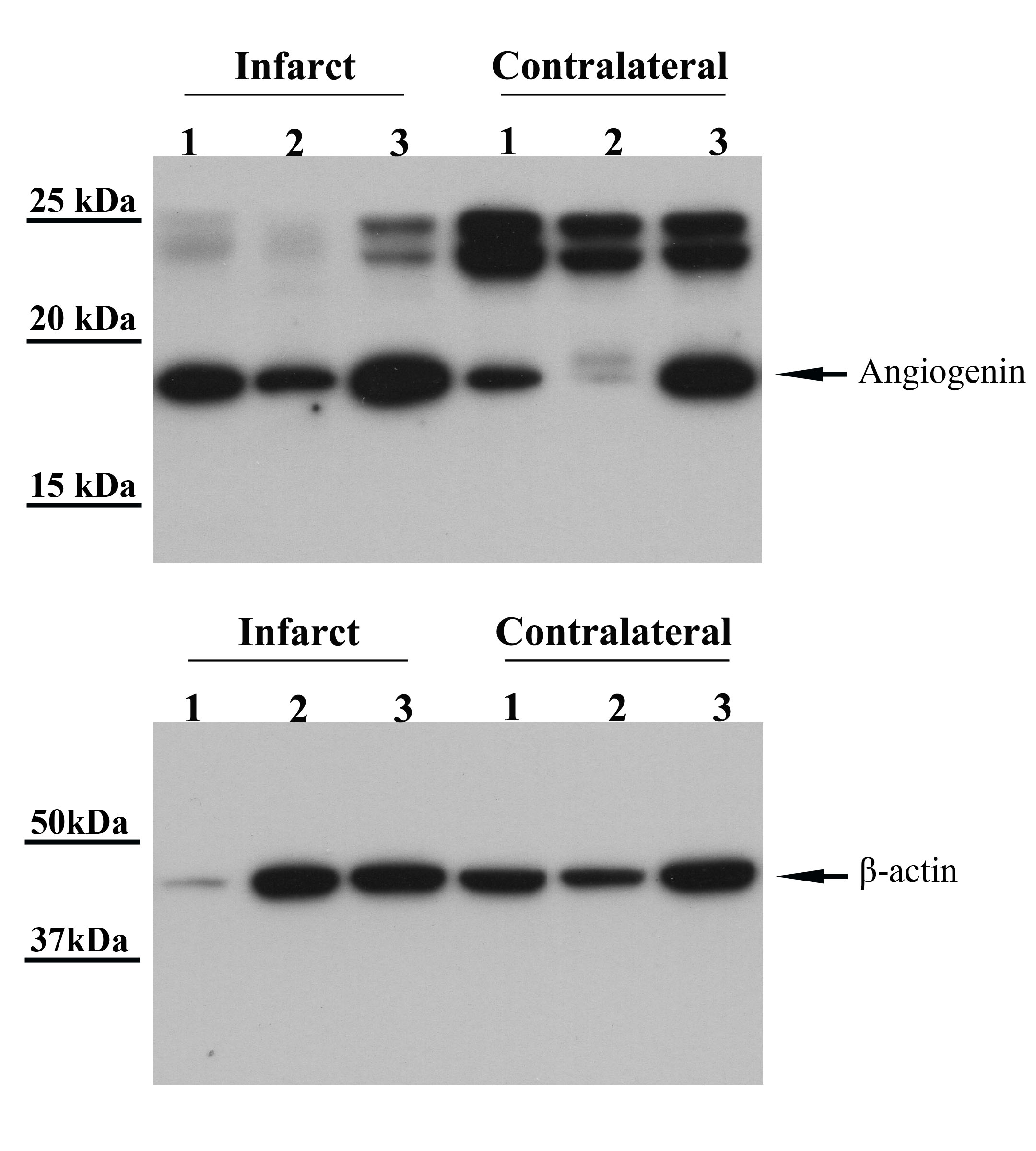

Supplement: Supplementary file 2 [file Image_1.JPEG]

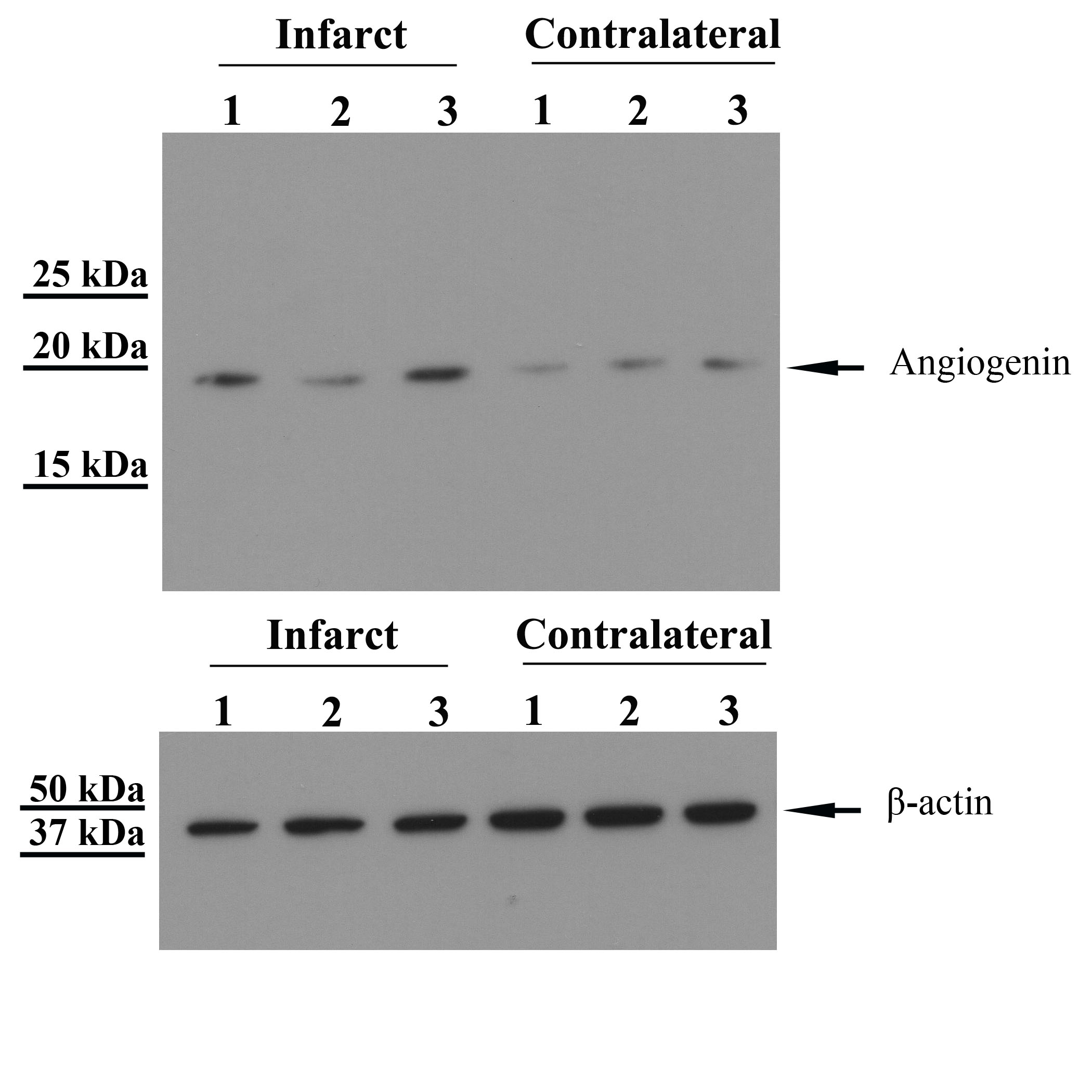

Supplement: Supplementary file 3 [file Image_2.JPEG]
